# Changes in physical activity level of adolescents and its relationship with mental health during regular COVID‐19 prevention and control

**DOI:** 10.1002/brb3.3116

**Published:** 2023-06-16

**Authors:** Ru‐bao Dong, Kai‐yun Dou

**Affiliations:** ^1^ School of Physical Education Guizhou Normal University Guiyan China

**Keywords:** physical activity, psychological health, regular epidemic prevention, SARS‐CoV‐2, teenagers

## Abstract

**Purpose:**

This study aimed to analyze the impact of regular prevention and control of COVID‐19 on the physical activity level (PAL) of adolescents and the relationship between PAL and mental health.

**Methods:**

Using the convenience sampling method, two stages of the on‐site cross‐sectional investigation were conducted in 11 middle schools in Guiyang City in China. There were 1132 participants who completed the Physical Activity Questionnaire for old children (PAQ‐C) in October 2020, and 1503 participants who completed the PAQ‐C and Mental Health Inventory of Middle‐school students (MMHI‐60) in October 2021. All participants reported their demographic data. Descriptive, quantitative statistics were used for data analysis. One‐way ANOVA was used to explore associations between PAL and mental health.

**Results:**

The results of statistical analysis showed a gradual yearly increase in the PAL of teenagers, and the PAL of male junior middle school students has a significant increase (*p* < .05); while that of adolescents in Grade 10 significantly decreased (*p* < .001). There is a statistically significant correlation between adolescents’ mental health (except for anxiety) and PAL (*p* < .05). The overall abnormal rate of mental health was 27.9%; The PAL and the total mean score of mental health had a negative correlation (*p* < .001). There was a significant difference between mental health scores and corresponding PAL (*p* < .001). Furthermore, there are statistically significant differences in scores of mental health factors corresponding to varying PAL among junior high school students and male students (*p* < .05).

**Conclusions:**

The regular epidemic prevention and control measures had significant adverse effects on the PAL of girls and high school adolescents, especially Grade 10 adolescents. Improving adolescents’ PAL can promote their mental health. Interventions based on PAL slightly lower than the level recommended in the physical activity guidelines can bring significant mental health effects.

## Graphical abstract

This study assumed that the physical activity level has both direct and indirect effects on the mental health of adolescents through sleep duration and general self‐efficacy, and established the model of physical activity promote mental health of adolescents. Arrows indicate direct action and its direction; This figure shows that PAL can directly promote adolescents' mental health, while sleep duration and GSE can directly promote adolescents' mental health; PAL can directly increase adolescent sleep duration and improve general self‐efficacy; PAL can also indirectly promote adolescent mental health through sleep duration and self‐efficacy

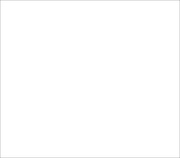

## 1 INTRODUCTION

According to the World Health Organization (WHO), 81% of adolescents aged 11−17 worldwide do not engage in enough physical activity (World Health, Organization), and one in seven (14%) adolescents aged 10−19 have experienced mental health problems (World Health, Organization). The coronavirus disease 2019 (COVID‐19) pandemic has had a severe impact on the mental health and well‐being of people worldwide (World Health, Organization); “isolation and social distance associated with COVID‐19 lead to a higher incidence of anxiety and depression” (Pearson, 2020). In the first year of the COVID‐19 pandemic, the physical activity time of children and adolescents showed a decline. Research shows that physical activity is related to positive mental health outcomes (Paterson et al., 2021). David Nieman suggests engaging in moderate‐intensity exercise during the COVID‐19 pandemic to enhance the immune system and eliminate the negative effects caused by aging, obesity, and lack of physical activity (Ainsworth & Li, 2020). Declining physical activity trends among adolescents are a key global health problem, affecting their cardiovascular metabolism and mental health (van Sluijs et al., 2021).

In April 2020, “guarding against imported cases and preventing a resurgence of the outbreak at home” and orderly “restart” of economic and social activities became the focus of the epidemic prevention and control work in China (武汉大学公共卫生治理研究课题组, 2020), marking the entry of China into the regular prevention and control of COVID‐19. Regular epidemic prevention and control measures, such as maintaining social distancing, have led to limited peer communication among adolescents and changes in the structure of daily life, which may have hidden adverse effects on the physical and mental health of adolescents (Oberle et al., 2010). Measures such as home quarantine and social distancing during an epidemic also cause a certain negative impact on physical exercise at all intensity levels (Ammar et al., 2020). Children and adolescents experienced decreased physical activity and increased sleep in the first year of the COVID‐19 pandemic (Ainsworth & Li, 2020). In the United States, the physical activity level (PAL) of people aged 3−18 years decreased significantly (Tulchin‐Francis et al., 2021). Therefore, the mental health of children and adolescents in the post‐epidemic era cannot be ignored (刘佳佳,阙建宇,张安易 & 陆林, 2021). Furthermore, physical exercise plays a more decisive role in an epidemic caused by a major infectious disease in that it can alleviate psychological stress and stabilize the emotional state, improve the body's immunity, and has a placebo effect (娄虎, & 颜军, 2020). Physical exercise can effectively resist and reduce the negative impact of COVID‐19 on people's physical and mental health (聂应军,谭珍科,马园艳 & 赵高彩, 2021) and plays a significant role in regulating the psychological effect of the COVID‐19 pandemic on primary and secondary school students (黄艳,王亭亭,黄金岩 & 徐思盈, 2021). Although physical exercise effectively counteracts and reduces the negative impact of COVID‐19 on physical and mental health (聂应军,谭珍科,马园艳 & 赵高彩, 2021), further research is required to assess the effects of different levels of physical activity on mental health during the COVID‐19 pandemic (Abdelbasset et al., 2021). Therefore, studying the adolescent physical activity change trend and mental health status and their relationship in the post‐epidemic era can not only help understand the impact of COVID‐19 on adolescent physical activity and enrich the research on the relationship between adolescent PAL and mental health but also acts as a theoretical reference for adolescent physical activity and mental health intervention.

## 2 RESEARCH DESIGN

### 2.1 Sample size

The sample size was calculated according to the following predictive equation (吴明隆, 2010):

$$n^{3}{\left(k/\alpha \right)}^{2}*p\left(1-p\right).$$

When *α* = 0.05, *p* = .5, then *k* = 1.96; The expected effective response rate of the questionnaire is 80%. Due to the fact that adolescents aged 12–18 are actually divided into two qualitatively different groups: middle school students and high school students, the sample size for each group is ≥ 482, and the total sample size is ≥ 964. The sample size for both stages is≥ 964, thus meeting the sampling requirements.

### 2.2 Measuring tools

The self‐made questionnaire, Physical Activity Questionnaire for Children (PAQ‐C) (郭强, 2016), and Mental Health Inventory of Middle‐school students (MMHI‐60) (郭强, 2016) were used for analysis. The reason for using PAQ‐C is that primary and secondary schools in China have 1–2 sports activities with a duration ≥25 min on school days. PAQ‐C was rated on a 5‐point Likert scale, and the Chinese version of the scale had good reliability and validity (王极盛, 李焰, & 赫尔实, 1997; 郭强, 2016). MMHI‐60 has 60 items, which are divided into 10 subscales using a 5‐point Likert scale, with the average score of 2−2.99 indicating the existence of mild problems, 3−3.99 indicating moderate problems, and 4−5 indicating serious problems. A total mean score of more than 2 indicated overall mental health problems. The scale had good reliability and validity (徐立峰, 2009; 陈小萍,安龙 & 马莹, 2020). In this study, the Kaiser−Meyer−Olkin construct validity coefficients for PAQ‐C and MSSMHS were .873 and .981, respectively, and Cronbach's α of internal consistency was .802 and .971, respectively.

The Physical Activity Questionnaire for Adolescents (PAQ‐A)/PAQ‐C classification standard of Chen et al. (2008) was adopted to divide the PAL of Chinese children and adolescents into three categories: vigorous, medium, and low. Vigorous physical activity level (VPAL) refers to reaching the recommended level of adolescent physical activity. Medium physical activity level (MPAL) is the level of physical activity slightly below the recommended guidelines. Low physical activity level (LPAL) stands for physical inactivity. Furthermore, based on the MMHI‐60 scores reflecting the overall psychological health status, adolescents were divided into two categories: the normal group and the abnormal group. Since the sample size of the more severe and above grades was extremely small, the status of each mental health factor was divided into four grades: normal, mild, moderate, and more severe and above.

### 2.3 Research subjects

The study was approved by the Academic Ethics Committee of the School of Physical Education of Guizhou Normal University. The questionnaire survey was conducted by random and convenient sampling after obtaining oral consent from the principals of relevant schools and the parents of students. Investigators distributed and collected questionnaires on‐site in the classroom on rainy days when it was not suitable to go outdoors and attend physical education classes. The participants read and signed the informed consent form before completing the questionnaire. The survey was conducted in 2020 and late October 2021 in three senior high schools and eight junior high schools in Guiyang City, Guizhou Province. Data were collected from one class/grade of junior high school students and two classes/grades of senior high school students. The survey in 2021 added a mental health component. The distribution, average age, and PAL of the survey subjects are shown in Table 1.

**TABLE 1:** Grades and gender distribution of the respondents.

Time	7	8	9	10	11	12	Female	Male	Age	PAL	Total
2020	292	198	149	178	152	163	613	519	14.81 ± 1.78	2.47 ± 0.73	1132
2021	331	302	269	234	220	147	742	761	14.94 ± 1.76	2.49 ± 0.67	1503

### 2.4 Statistical tools and methods

Statistical significance was set at *p* < .05. All statistical analyses were conducted using the statistical package for the social sciences (SPSS‐26.0 software, Armonk, NY, USA).

## 3 RESULTS

### 3.1 Changes in adolescent PAL

The average PAL of adolescents between 2020 and 2021 ranged from 2.47 to 2.49; the PAL in 2021 was slightly higher, but the difference was not statistically significant. The PAL of both junior and senior high school teenagers slightly improved. Regarding the grade, except for a significant decrease in the PAL of adolescents in Grade 10 (95% CI = .125,.403; *F* = 15.075, *p* < .001), the PAL of adolescents in other grades increased slightly. Adolescents in Grade 8 had the highest PAL, while adolescents in Grades 9 and 12 had the lowest PAL in junior and senior high school, respectively. From the perspective of gender, the PAL of male adolescents increased slightly, while female adolescents showed a slight decline. Furthermore, only male students in junior high school showed significant improvement in PAL (95% CI = −.198, −.002; *F* = 3.978, *p* = .046); both female and male high school students showed a slight decline in PAL (see Table 2).

**TABLE 2:** Statistical description and ANOVA of adolescent physical activity level.

						95% CI for mean		ANOVA of PAL	
Type	Time	*N*	Mean	Std. deviation	Std. error	Lower	Upper	F	P
Total	2020	1132	2.469	.734	.022	2.426	2.512	.744	.389
	2021	1503	2.493	.667	.017	2.459	2.526		
Female	2020	613	2.344	.657	.027	2.292	2.396	.769	.381
	2021	742	2.314	.595	.022	2.271	2.357		
Male	2020	519	2.616	.792	.035	2.548	2.685	1.448	.229
	2021	761	2.667	.687	.025	2.618	2.715		
Junior	2020	639	2.634	.652	.026	2.584	2.685	.107	.744
	2021	902	2.645	.647	.022	2.603	2.688		
Senior	2020	493	2.255	.779	.035	2.186	2.323	.043	.836
	2021	601	2.263	.630	.026	2.213	2.314		
Female of J	2020	333	2.554	.630	.035	2.486	2.622	3.281	.070
	2021	461	2.477	.567	.026	2.425	2.529		
Female of S	2020	280	2.094	.600	.036	2.024	2.165	.935	.334
	2021	281	2.047	.544	.032	1.984	2.111		
Male of J	2020	306	2.722	.665	.038	2.647	2.796	3.978	.046
	2021	441	2.822	.678	.032	2.758	2.885		
Male of S	2020	213	2.465	.925	.063	2.340	2.590	.033	.856
	2021	320	2.453	.641	.036	2.382	2.523		
7 grade	2020	292	2.666	.670	.039	2.588	2.743	.048	.827
	2021	331	2.677	.646	.036	2.607	2.747		
8 grade	2020	198	2.724	.590	.042	2.641	2.807	.149	.700
	2021	302	2.746	.636	.037	2.674	2.818		
9 grade	2020	149	2.454	.664	.054	2.347	2.562	.355	.552
	2021	269	2.493	.634	.039	2.417	2.569		
10 grade	2020	178	2.486	.789	.059	2.369	2.603	15.075	.000
	2021	234	2.222	.592	.039	2.145	2.298		
11 grade	2020	152	2.279	.779	.063	2.154	2.404	2.921	.088
	2021	220	2.406	.649	.044	2.320	2.493		
12 grade	2020	163	1.979	.679	.053	1.874	2.084	3.396	.066
	2021	147	2.116	.622	.051	2.014	2.217		

Abbreviations: Female/male of J = the girl/boy of junior high school student; Female/male of S = the girl/boy of senior high school student; Junior = junior high school student; Senior = senior high school student.

### 3.2 Adolescent PAL and mental health

#### 3.2.1 Descriptive statistics and correlation analysis

The total average score of adolescent mental health was 1.72 ± 0.63, and the score of each psychological factor ranged from 1.59 to 1.91, which was lower than the overall critical value of 2, indicating that most adolescents had good mental health. The overall level of mental health was at an abnormal rate of 28%. A higher value of the total mean score of adolescent mental health and the mean score of all individual factors indicate higher abnormal rates. The abnormal rate of the health status of each psychological factor was between 24.4% and 42.3%: obsessive‐compulsive symptoms and emotional imbalance had an abnormal rate of over 40%; anxiety, learning pressure, and depression had an abnormal rate of over 30%; psychological imbalance was the only factor with an abnormal rate slightly lower than 25%.

The correlation coefficient between adolescent PAL and the total mean score of mental health and scores of each psychological factor ranged from −0.86 to −1.43. Except for anxiety (*p* = .001), all other nine psychological factors and the overall mean score of mental health and PAL had statistically significant *p* values (*p* < .001). Thus, high school teenagers’ PAL corresponded to decreased mental health scores and increased mental health levels (see Table 3).

**TABLE 3:** Pearson correlation test between scores of mental health and physical activity level.

	Maladjustment	Obsessive	Depression	Interpersonal	Enmity	Paranoia	Emotional	Learning	Anxiety	Psychological	TAS
Normal %	70.5	57.7	68.3	70.1	74.6	71	59.5	67.9	65.4	75.6	72.1
Abnormal %	29.5	42.3	31.7	29.9	25.4	29	40.5	32.1	34.6	24.4	27.9
Mean	1.67	1.91	1.72	1.69	1.59	1.66	1.89	1.73	1.79	1.59	1.72
St. deviation	.67	.68	.79	.72	.71	.70	.77	.78	.83	.69	.63
Correlation	−.107	−.101	−.123	−.110	−.131	−.143	−.101	−.112	−.086	−.177	−.138
*p*	.000	.000	.000	.000	.000	.000	.000	.000	.001	.000	.000

TAS = total average score, Obsessive = obsessive‐compulsive symptoms , Interpersonal = interpersonal relationship, Emotional = emotional imbalance, Learning = learning pressure, Psychological = psychological imbalance (the same below).

#### 3.2.2 Relationship between adolescent PAL and mental health

The mean score of overall mental health and the scores of all 10 psychological factors decreased with the increase in PAL; that is, the mental health score corresponding to LPAL was the highest, and that corresponding to VPAL was the lowest (except for the obsessive‐compulsive symptoms factor). The difference in the correlation scores of the psychological factors corresponding to LPAL and MPAL ranged between 0.13 and 0.21, and those corresponding to MPAL and VPAL ranged between −0.02 and 0.1. The health scores of the psychological factors, emotional imbalance, and obsessive‐compulsive symptoms, corresponding to LPAL were equal to and exceeded the critical value of 2, respectively. Thus, adolescents with LPAL were more likely to have emotional imbalance and obsessive‐compulsive symptoms.

Furthermore, except for hostility, paranoia, and learning pressure, scores of other mental health factors were lower for high school students than for junior school students. Thus, compared with junior school students, high school students were more likely to suffer from hostility, paranoia, and learning pressure. Mental health scores of junior high school teenagers gradually decreased with the increase in PAL; that is, junior high school teenagers’ PAL was positively correlated with their mental health level. For high school adolescents, mental health corresponding to LPAL had the highest score, and MPAL had the lowest score. Furthermore, health scores of learning pressure and psychological imbalance for MPAL were equal to those for VPAL, while the total mean score of mental health and scores of the other eight factors were higher for VPAL than for MPAL. Thus, the mental health level of high school adolescents first increased gradually with the improvement in PAL; the mental health level was the highest for MPAL (slightly lower than the recommended PAL), and finally, the mental health level gradually decreased with the improvement in PAL.

From the perspective of gender, except for the equal scores for maladjustment, mental health scores for all other factors were higher for female students than for male students; that is, male students were more likely to have maladjustment problems than female students, while female students were more likely to have issues with overall mental health and the other nine mental health factors (see Table 4).

**TABLE 4:** Descriptive statistics of physical activity level and scores of mental health of adolescents.

Stage	PAL		TAS	Maladjustment	Obsessive	Depression	Interpersonal	Enmity	Paranoia	Emotional	Learning	Anxiety	Psychological
Junior	Low (148)	Mean	1.93	1.82	2.12	1.97	1.91	1.82	1.87	2.08	1.90	1.98	1.86
		Std. deviation	.63	.68	.69	.78	.72	.74	.73	.80	.79	.84	.81
	Middle (505)	Mean	1.71	1.65	1.91	1.71	1.70	1.57	1.63	1.90	1.71	1.81	1.55
		Std. deviation	.58	.63	.63	.79	.71	.68	.64	.71	.72	.81	.66
	High (249)	Mean	1.65	1.61	1.90	1.63	1.64	1.48	1.56	1.83	1.66	1.75	1.43
		Std. deviation	.54	.58	.67	.69	.66	.62	.62	.71	.70	.78	.57
	Subtotal (902)	Mean	1.73	1.67	1.94	1.73	1.71	1.59	1.65	1.91	1.73	1.82	1.57
		Std. deviation	.59	.63	.66	.77	.70	.69	.66	.73	.73	.81	.68
Senior	Low (226)	Mean	1.79	1.75	1.95	1.78	1.72	1.64	1.75	1.95	1.80	1.82	1.71
		Std. deviation	.73	.77	.73	.88	.81	.79	.83	.85	.87	.90	.80
	Middle (302)	Mean	1.66	1.62	1.80	1.64	1.62	1.56	1.62	1.78	1.71	1.69	1.57
		Std. deviation	.64	.69	.67	.77	.70	.68	.71	.79	.83	.80	.65
	High (73)	Mean	1.71	1.65	1.88	1.73	1.66	1.64	1.67	1.82	1.71	1.77	1.57
		Std. deviation	.71	.70	.72	.87	.70	.85	.74	.85	.86	.92	.66
	Subtotal (601)	Mean	1.71	1.67	1.86	1.70	1.67	1.60	1.68	1.85	1.74	1.75	1.62
		Std. deviation	.69	.73	.70	.83	.74	.75	.76	.83	.85	.85	.72
Male	Low (137)	Mean	1.89	1.84	2.06	1.93	1.82	1.74	1.84	2.05	1.90	1.94	1.77
		Std. deviation	.75	.78	.77	.93	.82	.84	.82	.89	.91	.93	.81
	Middle (394)	Mean	1.68	1.63	1.87	1.66	1.64	1.56	1.61	1.84	1.68	1.73	1.58
		Std. deviation	.62	.66	.67	.77	.69	.68	.67	.76	.75	.81	.68
	High (230)	Mean	1.66	1.64	1.89	1.63	1.64	1.51	1.60	1.84	1.69	1.73	1.47
		Std. deviation	.60	.60	.69	.73	.69	.68	.68	.77	.78	.82	.60
	Subtotal (761)	Mean	1.71	1.67	1.91	1.70	1.67	1.58	1.65	1.88	1.72	1.77	1.58
		Std. deviation	.64	.67	.70	.80	.72	.72	.70	.79	.79	.84	.69
Female	Low (237)	Mean	1.82	1.74	1.99	1.80	1.78	1.69	1.77	1.97	1.80	1.85	1.77
		Std. deviation	.66	.71	.70	.79	.75	.74	.78	.80	.80	.84	.81
	Middle (413)	Mean	1.71	1.64	1.87	1.71	1.70	1.58	1.65	1.87	1.74	1.80	1.53
		Std. deviation	.60	.65	.62	.79	.72	.69	.67	.72	.78	.81	.64
	High (92)	Mean	1.66	1.58	1.90	1.70	1.65	1.54	1.55	1.78	1.62	1.80	1.42
		Std. deviation	.53	.62	.66	.75	.61	.70	.57	.67	.62	.81	.58
	Subtotal (742)	Mean	1.74	1.67	1.91	1.74	1.72	1.61	1.68	1.89	1.74	1.81	1.60
		Std. deviation	.61	.66	.65	.79	.72	.71	.70	.75	.77	.82	.70
Total	Low (374)	Mean	1.84	1.78	2.02	1.85	1.80	1.71	1.80	2.00	1.84	1.88	1.77
		Std. deviation	.70	.74	.72	.85	.78	.78	.79	.83	.84	.88	.81
	Middle (807)	Mean	1.69	1.64	1.87	1.68	1.67	1.57	1.63	1.86	1.71	1.76	1.56
		Std. deviation	.61	.65	.65	.78	.70	.68	.67	.74	.76	.81	.66
	High (322)	Mean	1.66	1.62	1.89	1.65	1.64	1.52	1.58	1.83	1.67	1.75	1.46
		Std. deviation	.58	.61	.68	.74	.67	.68	.65	.75	.74	.81	.60

The results of one‐way ANOVA showed a statistically significant correlation between adolescents’ overall mental health and other health factors (except for anxiety which did not show a significant correlation) with different levels of physical activity (*p* < .05). Furthermore, there were statistically significant differences in scores of mental health factors corresponding to varying levels of physical activity between junior high school students and male students (*p* < .05). From the perspective of gender, male students had significant differences in mental health scores corresponding to different levels of physical activity. For female students, only the total mean score of mental health, paranoia, and psychological imbalance showed significant differences for varying levels of physical activity. There were significant differences in mental health scores corresponding to different levels of physical activity among junior high school students. However, only obsessive‐compulsive symptoms showed a significant difference in scores corresponding to varying levels of physical activity among senior high school students. This indicates that mental health scores corresponding to different levels of physical activity differ by gender and age (see Table 5).

**TABLE 5:** Summary of ANOVA between mental health scores and physical activity levels of different types of groups.

	Junior high school students		Senior high school students		Male		Female		Total	
	*F*	Sig.	*F*	Sig.	*F*	Sig.	*F*	Sig.	*F*	Sig.
TAS	11.705	.000	2.106	.123	6.478	.002	3.301	.037	9.391	.000
Maladjustment	5.667	.004	2.041	.131	5.310	.005	2.550	.079	6.722	.001
Obsessive	6.909	.001	3.219	.041	4.218	.015	2.904	.055	6.740	.001
Depression	9.552	.000	1.760	.173	7.480	.001	1.252	.287	7.354	.001
Interpersonal	7.265	.001	1.242	.290	3.436	.033	1.604	.202	5.174	.006
Enmity	12.107	.000	0.762	.467	5.043	.007	2.281	.103	7.390	.001
Paranoia	11.403	.000	1.838	.160	6.735	.001	4.015	.018	10.121	.000
Emotional	5.797	.003	2.738	.066	3.849	.022	2.601	.075	5.830	.003
Learning	5.048	.007	0.708	.493	4.393	.013	1.844	.159	4.558	.011
Anxiety	3.987	.019	1.352	.259	3.411	.034	0.316	.729	2.967	.052
Psychological	19.794	.000	2.775	.063	8.081	.000	11.953	.000	19.476	.000

## 4 DISCUSSION

### 4.1 Adolescent PAL

Based on the PAQ‐C/PAQ‐A classification (Benítez‐Porres et al., 2016; Chen et al., 2008; Dan et al., 2007), the PAL of adolescents in the post‐epidemic period was found to be in a medium and low state, which was consistent with the “low high‐intensity physical activity level among children and adolescents in China” (张曌华,李红娟,桂春燕 & 张婷, 2019) in the pre‐epidemic period. Furthermore, adolescent PAL in this study conforms to the reports that “adolescent physical activity level gradually decreases with age” (Voss et al., 2013). The PAL of female students was lower than that of male students, which is consistent with the findings of previous studies (吴慧攀,张明,尹小俭,李佳威,邓婷,张祥… & 康栩烨, 2022; 葛志刚,杨岚岚 & 陈军, 2021). The adolescent PAL in this study, conducted in Guizhou Province, was lower than that of adolescents in Beijing (王极盛, 李焰, & 赫尔实, 1997), the national average (张曌华,李红娟,桂春燕 & 张婷, 2019) and the average level of adolescent physical activity in multiple provinces and cities (郭强, 2016), but was higher than that of 12‐ to 18‐year‐old adolescents in Guizhou Province in 2020 (孙涛涛, 2021) before the COVID‐19 pandemic. Therefore, although “the restrictions imposed by the pandemic may hurt physical activity” (Colabianchi et al., 2016; Sallis et al., 2000), leading to reduced physical activity time for children and adolescents (Oberle et al., 2010), and measures such as home isolation and social distancing during the epidemic also harmed physical exercise at all levels of intensity (Ammar et al., 2020).

Maybe most people decreased their PA since COVID‐19 restrictions (Caputo & Reichert, 2020; Stockwell et al., 2021).

Because the government has adopted the strictest normalized epidemic prevention and control measures, such as “social distancing,”“ lock‐downs,” “closure of sports halls and gymnasiums” to mitigate the spread of COVID‐19 in China. These approaches restrict the mobility, daily activities (Ammar et al., 2020; Ammar et al., 2020; Trabelsi et al., 2021), and social interactions of the individuals (Ammar et al., 2020; Ammar et al., 2021). So, the reduction in PA time and step counts was accompanied by increased behavioral stress during COVID‐19 lockdown (Dergaa et al., 2022).

The findings in this study showed that adolescents in China adapted to the regular prevention and control of COVID‐19 and their PAL gradually increased, returning to the level before the epidemic. In particular, junior high school boys significantly improved their level of physical activity.

### 4.2 Adolescent mental health level

Symptoms of psychological distress and disorder have been widely reported in people under quarantine during the COVID‐19 pandemic (Trabelsi et al., 2021). Two studies indicate that the COVID‐19 outbreak engendered anxiety, depression, sleep disturbances, and other psychological issues in China (Qiu et al., 2020; Wang et al., 2020). The abnormal rate of overall mental health among adolescents was 27.9%, higher than the average reported by WHO. The abnormal rate of each mental health factor ranged from 24.4% to 42.3%, slightly lower than the rate reported by studies (Zhou et al., 2020; 肖三蓉,王秋馨 & 徐光兴, 2021) at the early stage of the COVID‐19 outbreak and somewhat higher than that reported by other studies (丁文清,周苗 & 宋菲, 2017; 林红,黄乾坤 & 佟圣丽, 2022; 梁汀, 2021; 陈丹,权治行,艾梦瑶,宗春山 & 许建农, 2020). However, the abnormal rate of each mental health indicator ranged from 10% to 60% (肖三蓉,王秋馨 & 徐光兴, 2021) in the same type of research results, which might be the result of gradual improvement in physical activity because “physical exercise helps to improve the mental health of children” (傅晰, 2022). The abnormal rate of obsessive‐compulsive symptoms was the highest, reflecting the prevention and control measures taken by adolescents, such as washing hands frequently and maintaining social distancing.

There was conclusive evidence to support the potential negative impact of the pandemic on adolescent mental health. Stressful life events, extended home confinement, worry, overuse of the internet, and social media are factors that could influence the mental health of adolescents during this pandemic (Jones et al., 2021). Globally, adolescents of varying backgrounds experience higher rates of anxiety, stress (Trabelsi et al., 2021), and depression (Duan et al., 2020; Ellis et al., 2020; Qi et al., 2020; Tee et al., 2020; Zhang et al., 2020) due to the pandemic.

In China, due to the heavy learning tasks of middle school students, parents and children lack sufficient communication in their daily lives. To some extent, this situation has been improved and has also alleviated some of the psychological burden on teenagers during the epidemic. Because studies have shown that the implementation of social support leads to positive mental health outcomes (Qi et al., 2020). The positive benefits were related with coming in closer and having more family discussions between parents and their children during COVID‐19 home quarantining (Tang et al., 2021). Therefore, the changes in the mental health status of adolescents are relatively small during COVID‐19.

### 4.3 Adolescent physical activity and mental health

There was a positive correlation between adolescents’ PAL and mental health, and the differences between LPAL and various mental health factors (except anxiety) corresponding to MPAL or VPAL were statistically significant. In particular, emotional imbalance and obsessive‐compulsive symptoms corresponding to LPAL were in an abnormal state, indicating that good mental health effect could be achieved if the PAL was slightly lower than the level recommended by physical activity guidelines, and there was no significant difference between the mental health level and the recommended PAL. Research has shown that “moderate and above physical activity improved the mental health of adolescents, but no such association was found in low‐intensity physical activity” (Duan et al., 2020; Ellis et al., 2020). This study showed that mental health benefits brought by different levels of physical activity differed based on gender, age, and other psychological factors, indicating that PAL‐based interventions will have different effects in improving adolescents’ mental health.

Physical inactivity and reduced PA levels are associated with increased levels of anxiety during COVID‐19 pandemic in Canada (Qi et al., 2020; Zhang et al., 2020), Brazil (Doré et al., 2016; Tang et al., 2021), France, and Switzerland (Costigan et al., 2016). It has been shown that physical activity has a substantial positive effect on mental health (Tee et al., 2020). It may be a very effective way to improve the mental health and physical health through promoting the PAL of teenagers.

## 5 EPILOGUE

### 5.1 Conclusion

During the regular prevention and control of COVID‐19, the PAL of adolescents in China was generally medium and low. The findings of this study show that adolescents have adapted to the new normal, and their PAL has gradually returned to the level before the epidemic. Regular epidemic prevention and control measures mainly harm female and high school adolescents. The abnormal rate of adolescent mental health is within the normal range but is still high. The mental health level of senior high school students is higher than that of junior high school students, and of male teenagers is higher than that of female teenagers. There was a significant positive correlation between PAL and the mental health of teenagers. The findings suggest that a slightly lower PAL than the recommended level could significantly improve the mental health of teenagers. Improving VPAL can improve the overall mental health of middle school students and male students; however, there is a limited effect on the mental health of high school students and female students.

Therefore, if no distinction is made based on gender or age, mental health interventions for adolescents should consider a slightly lower level of physical activity than the recommended level. For male and junior high school adolescents, the recommended level of physical activity should be considered. For high school and female adolescents, PAL‐based mental health interventions have limited effect; nonetheless, if PAL‐based interventions are to be adopted, it is suggested to consider physical activities slightly lower than the recommended level.

### 5.2 Study limitations and future prospects

There are several limitations of this study. First, the data used in the research were obtained from self‐administered questionnaires. There might be memory bias in the data. Second, the samples in this study were all from Guizhou Province, with limited representation. Future research should expand the geographical scope of the study and adopt a longitudinal survey design to further analyze the relationship between PAL and the mental health of teenagers.

## AUTHOR CONTRIBUTIONS

Conceptualization, writing–review and editing, funding acquisition, investigation, methodology, software, formal analysis, Dong Ru‐bao; data curation, writing–original draft preparation, project administration, supervision, Dou Kai‐yun. All authors have read and agreed to the published version of the manuscript.

## ETHICS APPROVAL AND CONSENT TO PARTICIPATE

Before the start of this study, informed consent was obtained from all participants. This study was approved by the Ethics Committee at the School of Physical Education of the Guizhou Normal University of China. All methods were carried out in accordance with relevant guidelines and regulations.

We declare that none of the authors holds any financial interests or conflict of interest associated with the ChatGPT or NLM technologies discussed in this paper. None of the authors has collaborated or consulted with any individuals or organizations that have a financial or other interest in the ChatGPT or NLM technologies.^1^

## CONSENT FOR PUBLICATION

No individual or indemnifiable data are being published as part of this manuscript.

### PEER REVIEW

The peer review history for this article is available at: https://publons.com/publon/10.1002/brb3.3116.

## Data Availability

The data that support the findings of this study are available on request from the corresponding author. The data are not publicly available due to privacy or ethical restrictions.

## ENDNOTE

1 Dergaa, I., Chamari, K., Zmijewski, P., & Ben Saad, H. (2023). From human writing to artificial intelligence generated text: Examining the prospects and potential threats of ChatGPT in academic writing. *Biology of Sport, 40*(2), 615–622. https://doi.org/10.5114/biolsport.2023.125623
